# Color in Inlays

**Published:** 1906-07

**Authors:** C. H. Farrand


### COLOR IN INLAYS.

In the matter of color you will perhaps meet with more discouragements than in any other factor with which you have to do in the making of a porcelain inlay. In introducing the inlay to your practice, do not assure your patients that you can put in a filling that will look exactly like the natural tooth and that can not be seen, for you can not do so except in cases where you have been so fortunate as to have had a lucky accident, and where your perfect results are in no wise due to your skill. There are too many forces at work which you must be able to overcome to insure success in every case, and the least you can expect to do is to have your inlays pass unnoticed by the casual observer. If you attain this degree of perfection you are doing as much as, if not more, than the expert practitioner. It is a notable fact that the majority of inlays put in for clinical purposes are shown up in the cavity before being cemented. The result may be apparently perfect, but the addition of cement will in most cases tell a different story. If we had a transparent cement these apparent results could be readily obtained with but little effort on the part of the operator. Any color he might select which would at all approximate the color of the tooth would become so modified by the reflection of light through it from the tooth proper that it would escape the closest scrutiny. —*Dr. C. H. Farrand. Review.*

